# Adoptive cell therapy for cancer treatment

**DOI:** 10.1002/EXP.20210058

**Published:** 2023-07-02

**Authors:** Shi Du, Jingyue Yan, Yonger Xue, Yichen Zhong, Yizhou Dong

**Affiliations:** ^1^ Division of Pharmaceutics and Pharmacology College of Pharmacy Ohio State University Columbus USA; ^2^ Icahn Genomics Institute Precision Immunology Institute Department of Oncological Sciences Tisch Cancer Institute Friedman Brain Institute Icahn School of Medicine at Mount Sinai New York USA

**Keywords:** adoptive cell therapy, cancer immunotherapy, chimeric antigen receptors, dendritic cells, macrophages, natural killer cells, T‐cell receptors, tumor‐infiltrating lymphocytes

## Abstract

Adoptive cell therapy (ACT) is a rapidly growing anti‐cancer strategy that has shown promise in treating various cancer types. The concept of ACT involves activating patients’ own immune cells ex vivo and then transferring them back to the patients to recognize and eliminate cancer cells. Currently, the commonly used ACT includes tumor‐infiltrating lymphocytes (TILs), genetically engineered immune cells, and dendritic cells (DCs) vaccines. With the advancement of cell culture and genetic engineering techniques, ACT has been used in clinics to treat malignant hematological diseases and many new ACT‐based regimens are in different stages of clinical trials. Here, representative ACT approaches are introduced and the opportunities and challenges for clinical translation of ACT are discussed.

## Graphical abstract

This perspective describes multiple types of commonly used adoptive cell therapy with an emphasis on their clinical progress. In addition, the challenges and opportunities of adoptive cell therapy for future clinical translation are described from the authors' point of view.

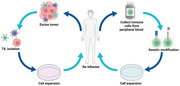

## 1 INTRODUCTION

**FIGURE 1:**
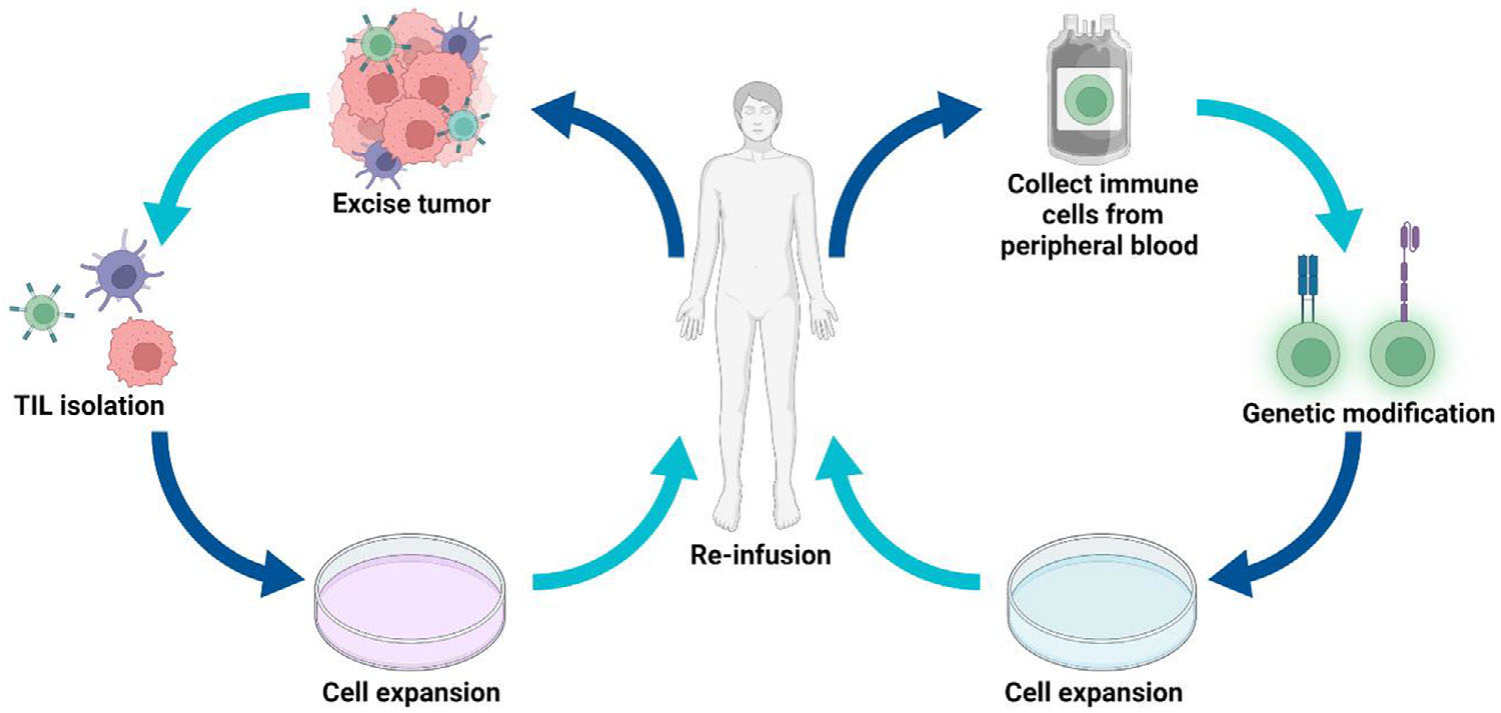
Illustration of adoptive cell therapy (ACT). Immune cells can either be collected from patients' excised tumors or peripheral blood. These cells are isolated, expanded, and modified to be given back into the hosts to exert anti‐tumor activity. Figure created using Biorender.com.

With the development of cancer immunotherapy, adoptive cell therapy (ACT) has emerged as an important therapeutic strategy against cancers.^[^ ^1^ ^]^ ACT generally refers to *ex vivo* engineering of patients’ own immune cells to strengthen the anti‐tumor immunity.^[^ ^2^ ^]^ ACT‐based cancer treatment is mainly composed of three steps: First, autologous immune cells are collected from the patient's peripheral blood or tumor tissues; Then, the cells are expanded and/or modified *ex vivo* to enhance the anti‐cancer activity; Finally, the modified cells are infused back to patients to mediate tumor regression (Figure 1). Compared with other cancer immunotherapy which relies on the host's intrinsic antitumor lymphocytes, ACT holds the advantages of sufficient quantities, modifiable functions, and durable responses.^[^ ^2^ ^]^ More importantly, as a personalized medicine, ACT can circumvent the concerns of individual differences in standard treatment options.^[^ ^1^ ^]^

**FIGURE 2:**
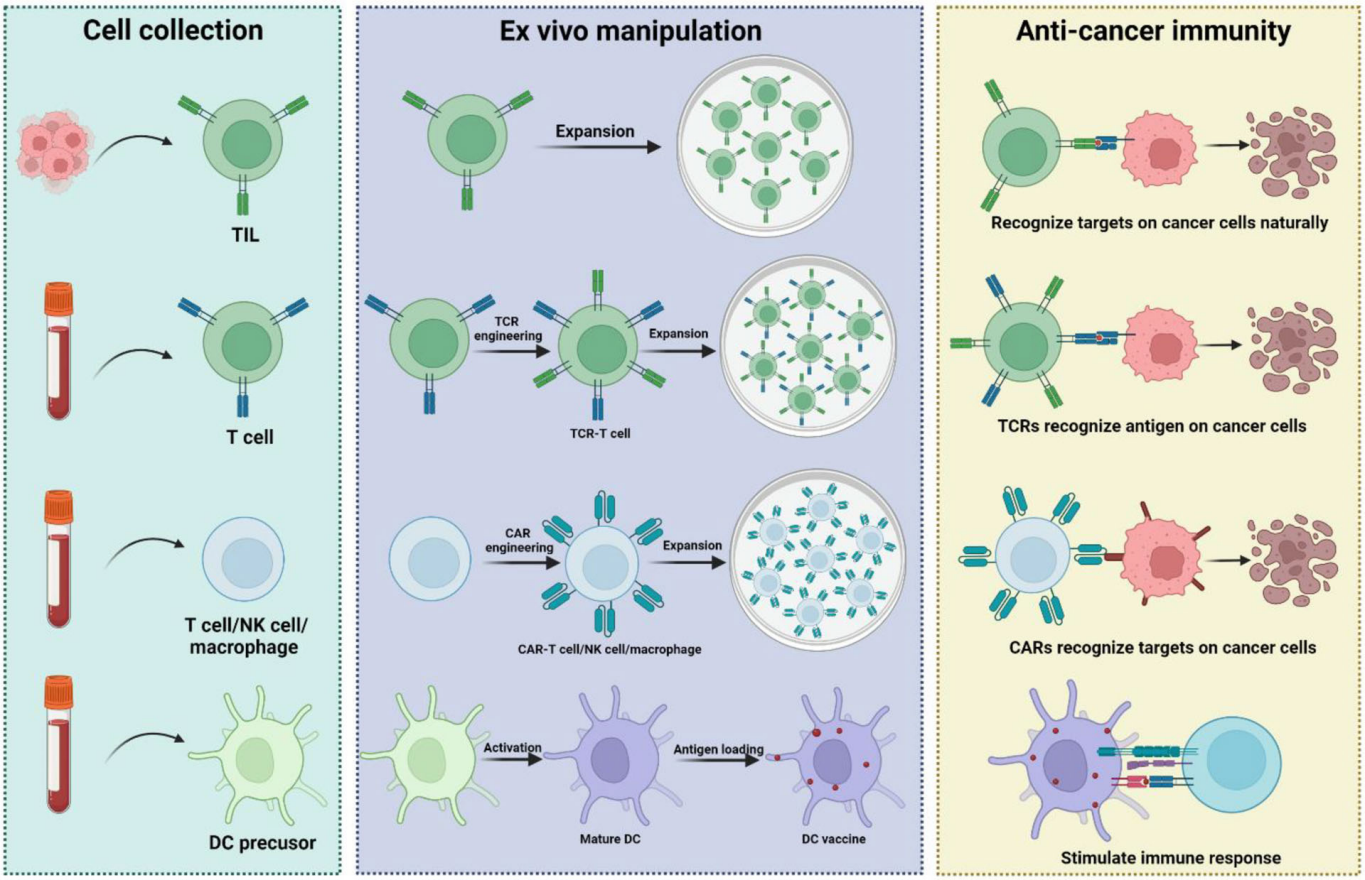
Diverse ACT platforms. ACTs mainly include Tumor‐infiltrating lymphocyte (TIL) therapy, T‐cell receptor (TCR) therapy, Chimeric antigen receptor (CAR) T cell/NK cell/macrophage therapy, and Dendritic cell (DC) therapy. Figure created using Biorender.com.

Over the past decades, ACT undergoes the transition from generalized lymphocytes to functional cancer‐specific lymphocytes.^[^ ^3^ ^]^ Early ACT therapy using tumor‐infiltrating lymphocytes (TILs) led to advances in metastasis melanoma treatment.^[^ ^4^ ^]^ However, TILs‐based ACT is only effective in partial patients. To broaden the application of ACT, genetic engineering technologies were developed to introduce T‐cell receptors (TCRs) and chimeric antigen receptors (CARs) to naturally occurring T lymphocytes.^[^ ^1^, ^3^ ^]^ Several types of genetically engineered T cells have been authorized for clinical use in treating hematologic malignancies.^[^ ^5^ ^]^ Moreover, this genetic engineering approach was extended to other subsets of immune cells, such as natural killer (NK) cells and macrophages.^[^ ^6^ ^]^ Besides direct tumor killing, another commonly used ACT is dendritic cell (DC)‐based vaccinations, which can induce a heightened anticancer immune response.^[^ ^7^ ^]^ Currently, ACT is constantly evolving and being evaluated, either alone or in combination with other immunotherapies in clinical trials.^[^ ^8^ ^]^ Notwithstanding recent progress, the tumor immunosuppressive microenvironment and cross‐reactive toxicity still restrict the clinical application of ACT.^[^ ^9^ ^]^ Therefore, various engineering strategies have been investigated to improve the specificity and safety of ACT. This perspective describes different types of commonly used ACT‐based treatment (Figure 2) with an emphasis on their clinical progress. We also highlight the challenges and opportunities of ACT for future clinical translation.

## 2 VARIOUS CELL TYPES FOR ADOPTIVE CELL THERAPY

### 2.1 Tumor‐infiltrating lymphocytes (TILs)

Initial studies of ACT can be traced back to the use of recombinant human cytokines interleukin‐2 (IL‐2), which provided the foundation of the *ex vivo* cultivation of non‐specific lymphocytes.^[^ ^10^ ^]^ In this context, lymphokine‐activated killer (LAK) cell therapy emerged for the treatment of metastatic melanoma.^[^ ^11^ ^]^ LAK cells are generally mixtures of NK cells and T lymphocytes with a tumor‐killing effect. Although pre‐clinical studies have indicated their anti‐tumor effectiveness, their therapeutic efficacy in clinical settings has not met expectations. Subsequent studies focused on lymphocytes with a more specific activity. In contrast to non‐specific LAKs, tumor‐infiltrating lymphocytes (TILs) derived from heterogeneous populations of lymphocytes in the resected tumors can recognize tumor‐specific antigens. After eliminating primary and metastatic tumors in mouse models, the anti‐tumor effects mediated by TILs were subsequently confirmed in melanoma patients.^[^ ^1^, ^12^ ^]^ However, these clinical trials showed that the infused TIL cells were rarely found in blood circulation just days after administration. To prolong the duration of TILs, lymphodepletion was introduced before cell transfer.^[^ ^13^ ^]^ Typically, lymphodepletion refers to total body irradiation or chemotherapy to kill endogenous immunosuppressive lymphocytes (lymphoid and myeloid populations). Both pre‐clinical and clinical studies showed that lymphodepletion improved the efficacy and duration of TIL treatment.^[^ ^14^ ^]^ For example, in a phase I/II clinical trial for metastatic melanoma, the researchers used a preparatory chemotherapy regimen to deplete the lymphocytes of the patients. Afterward, the patients were infused with TILs. The results of the study showed that 52% of the patients had an objective response to the treatment. Additionally, the median duration of response was 22.3 months, which suggests that the treatment was effective for a significant period.^[^ ^13a^ ^]^ This highlights the potential benefit of lymphodepletion in enhancing the effectiveness of TIL therapy.

The durable anticancer effect of TILs in metastatic melanoma has prompted attempts to treat other common cancers. Multiple clinical studies have been conducted to evaluate anti‐tumor efficiency of TILs in solid tumors including non‐small cell lung cancer (NSCLC), colorectal cancer (CRC), cervical cancer, and breast cancer.^[^ ^15^ ^]^ For example, in a phase II clinical trial of TIL therapy for cervical cancer, 44% of patients had an objective response to the therapy, and the median duration of response was 17.4 months.^[^ ^15a^ ^]^ However, the heterogeneity of solid tumor antigens greatly compromises the reactivity of TILs. Since TILs are only based on in vitro expansion of cells and do not introduce specific antigens, T cell subsets with low avidity to tumor antigens are not sufficient to stimulate efficient immune responses. To address this challenge, tumor mutant antigens were screened by whole‐exome and transcriptome sequencing, and TILs specific for these tumor antigens were isolated *ex vivo* for cell transfer.^[^ ^16^ ^]^ These personalized TILs showed significantly higher therapeutic effects than conventional TILs in patients with metastatic ovarian cancer.^[^ ^17^ ^]^ Although TILs‐based therapy has shown promising outcomes in the treatment of several types of cancer, it still has its limitations. First, the tedious and time‐consuming preparation process remains a challenge to produce TIL therapy, especially for patients with advanced cancers. In addition, the response rate of TIL treatment varies from 70% to 98%. This interpatient variability also hinders the clinical translation of TILs. Currently, a phase II clinical trial (NCT01174121) is evaluating the efficacy and safety of TILs on multiple tumors including the digestive tract, urothelial, breast, or ovarian/endometrial tumors. These clinical trials may provide new knowledge and improve ideas for TIL therapy in advanced cancer treatment.

### 2.2 TCRs modified T cells (TCR‐T cells)

TCRs, which are intricate surface proteins on T cells, have a crucial function in identifying and reacting to anomalous or foreign cells within the body. TCR‐T cells entail genetically modifying T cells to express TCRs capable of identifying particular antigens presented by tumor or abnormal cells. These modified T cells can then target and destroy the designated abnormal cells, leading to potential therapeutic benefits.^[^ ^18^ ^]^ Generally, TCRs consist of alpha and beta chains that can recognize and bind antigenic peptides presented by the major histocompatibility complex (MHC) molecules on the surface of cells. When a TCR recognizes an antigen, it triggers T cell activation and an immune response against the abnormal cells.^[^ ^19^ ^]^ The clinical efficacy of TCR‐T cell therapy has been demonstrated to treat melanoma and synovial sarcoma, and new tumor‐targeting antigens are constantly being identified and tested.^[^ ^20^ ^]^ To broaden the application of TCR‐T cell therapy, multiple tumor‐targeting antigens are identified and tested in the clinic.^[^ ^21^ ^]^ TCR‐targeted antigens can be broadly classified into two categories: Tumor‐associated antigens (TAAs) and tumor‐specific antigens (TSAs). TAAs are characterized by their overexpression in tumor cells including differentiation antigens as well as cancer‐testis antigens. In contrast, TSAs are derived from mutations that arise in tumor cells, as well as viral antigens that are produced by tumor viruses.

One challenge of TCR‐T cell therapy is that the heterogeneity of antigen expression often causes unexpected and severe side effects.^[^ ^22^ ^]^ For example, TCR‐T cells targeting the differentiation antigen MART‐1 can cause neurotoxicity and cardiotoxicity through cross‐reactivity with proteins on normal tissues.^[^ ^23^ ^]^ Fatal side effects were also observed in TCR‐T cells based therapy targeting the cancer/testis antigen (CT antigen) MAGE‐A3, which was caused by cross‐reactivity with MAGE‐A12 expressed in the brain.^[^ ^24^ ^]^ Overexpressed antigens are considered safer TAAs due to their higher level in tumor cells while lower expression in normal tissues. For example, WT1, an overexpressed antigen, is widely regarded as an excellent target for TCR‐T cells based therapy due to its high expression in a majority of acute myeloid leukemia (AML), acute lymphoblastic leukemia (ALL), and various solid tumors, while expressed only minimally in normal tissues.^[^ ^25^ ^]^ Another example with remarkable potency and low toxicity is CT antigen NY‐ESO‐1with differential expression between tumor tissues and normal tissues.^[^ ^26^ ^]^ Clinical studies have shown that NY‐ESO‐1‐specific TCR‐T cells are effective for synovial sarcoma, melanoma, and advanced myeloma without serious adverse events (NCT01343043). Recent clinical trials also demonstrated the efficacy of TCR‐T cells targeting human papillomavirus (HPV) E7 antigen to treat metastatic human papillomavirus‐associated epithelial cancers (NCT02858310). More recently, neoantigens associated with tumor mutations are becoming a hot spot for TCR targets.^[^ ^27^ ^]^ Promising neoantigens include KRAS G12D/G12V in pancreatic and colorectal cancer and PIK3CA H1047L in metastatic breast cancer.^[^ ^28^ ^]^ Notably, most neoantigens are derived from random mutations that are typically not shared among patients. Thus, identification of neoantigens might require sequencing the entire genome of each tumor to predict the appropriate antigen candidates.^[^ ^29^ ^]^

In addition to the antigen selection, TCR engineering is another critical step determining the activity and compliance of TCR‐T cell therapy. One challenge of TCR engineering is the correct pairing of TCR α/β chains. Mispairing of introduced TCRs and endogenous TCRs could impact the efficacy and cause graft‐versus‐host‐disease toxicity (GVHD).^[^ ^30^ ^]^ Therefore, several strategies have been developed to avoid the mispairing of TCRs. One representative method is replacing constant regions of human TCRs with murine regions. Though there is concern that foreign murine TCRs may trigger an immune response, clinical studies showed immunogenicity of these recombinant TCRs is negligible.^[^ ^31^ ^]^ Some other preclinical studies promoted TCR α/β dimerization by substituting certain residues or tuning the structure of TCRs.^[^ ^32^ ^]^ Common approaches include introduction of disulfide bonds, transmembrane hydrophobic substitutions, introduction of residues mediating TCR α/β dimerization, construction of single‐chain TCRs and knock‐out of endogenous TCRs.^[^ ^33^ ^]^ Another challenge of TCR engineering is the MHC restriction. TCR targets are limited to HLA‐A*02:01 allele which accounts for a proportion of people. Therefore, MHC‐independent TCR T cells were explored. Some naturally occurring TCRs from CD1‐restricted T cells or monomorphic MHC class I‐related protein (MR1)‐restricted T cells can recognize lipids or proteins on the tumors in an MHC‐independent manner.^[^ ^34^ ^]^ Another strategy is fusing the antibody Fab domain with the effector domain from γ/δ TCR to the construct antibody‐TCR (AbTCR). For example, AbTCR targeting CD19 can activate endogenous T cells and modulate signaling pathways without co‐stimulation. In a xenograft mouse model, anti‐CD19‐AbTCR‐T cells released fewer inflammatory cytokines than anti‐CD19‐CAR‐T cells, indicating its potential as a safer and more effective TCR‐T therapy.^[^ ^35^ ^]^

Although TCR‐T cell therapy displayed effectiveness in the treatment of some forms of blood cancer, its efficacy in treating solid tumors has been limited. Researchers are exploring various strategies to improve the effectiveness of TCR‐T cell therapy in solid tumors such as identifying new cancer antigens and investigating the combination of TCR‐T cell therapy with other cancer treatments. Moreover, ongoing research is critical to developing standardized methods for manufacturing and delivery of TCR‐T cells, as well as optimizing the dosing and administration of the therapy.

### 2.3 Chimeric antigen receptor T‐cells (CAR T cells)

**FIGURE 3:**
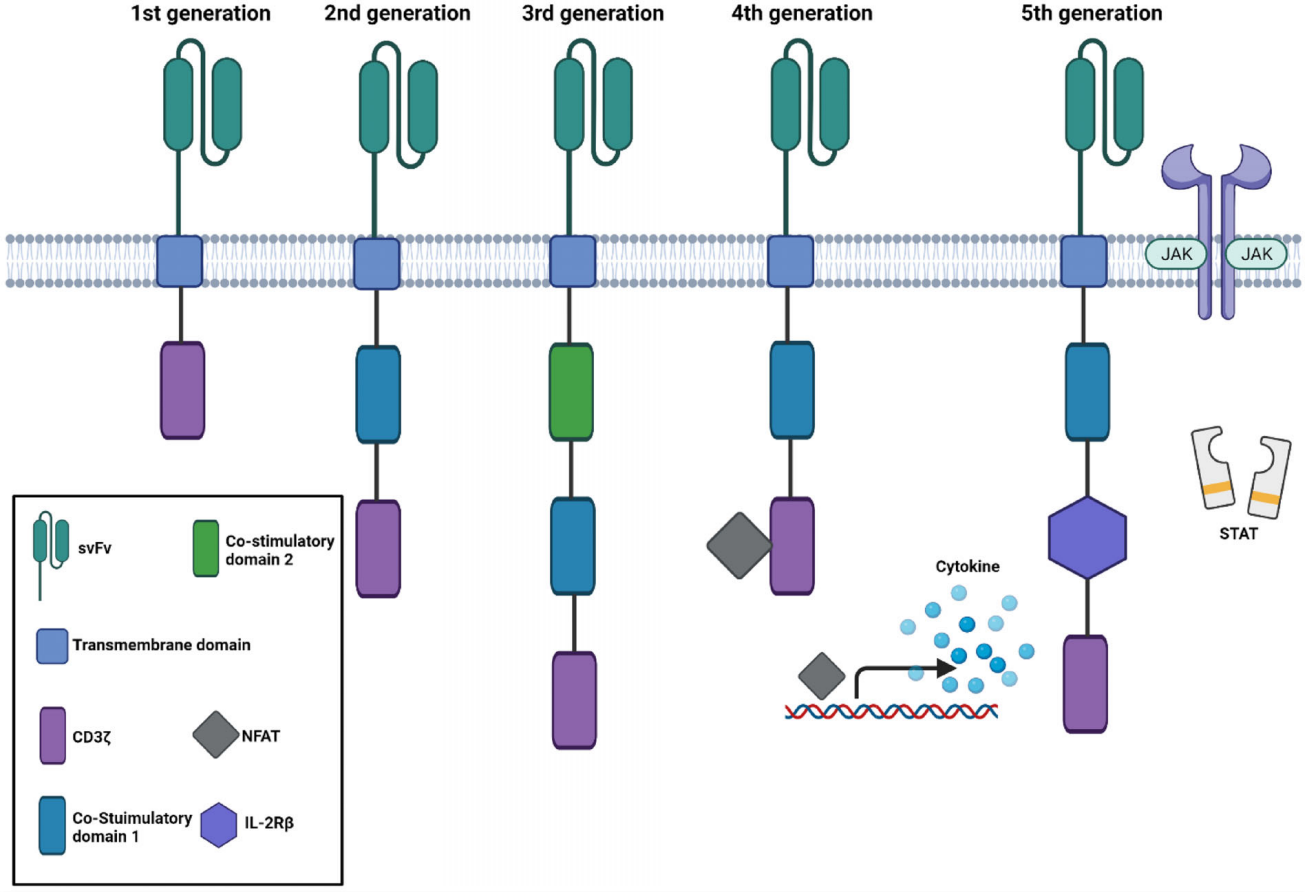
The evolution of CARs. The first generation contains CD3ζ as a stimulatory domain for T cell activation. The second‐generation and third‐generation CARs include one or multiple costimulatory domains. The fourth‐generation CARs can indelibly produce specific cytokines such as IL‐12 or IL‐2 driven by the nuclear factor of activated T‐cells (NFAT). The fifth‐generation CARs integrate an IL‐2Rβ domain which can induce antigen‐dependent activation based on JAK‐STAT pathway. Figure created using Biorender.com.

The Introduction of CARs is another critical strategy to improve the specificity and activity of native T cells. With the ability to recognize antigens or proteins on cancer cells without MHC restriction, CAR‐T therapy effectively circumvents immune escape caused by down‐regulation of MHC‐associated antigens on cancerous cells. Based on the structure of CARs, CAR‐T cells are typically divided into five generations (Figure 3). The first‐generation CARs mimic the endogenous TCRs consisting of an extracellular single‐chain variable fragment (scFv) and an intracellular activation domain (CD3ζ). However, due to the rapid cell depletion and insufficient cytokine secretion, the in vivo anti‐tumor activity of the first‐generation CARs is greatly limited. The second‐generation of CARs introduce a costimulatory molecule in the intracellular region, which enhances the T cell activation and persistence. Currently, the CAR‐T therapies approved by the US Food and Drug Administration (FDA) are based on this generation of CARs. The third‐generation CARs are featured by the addition of multiple co‐stimulatory signaling domains (e.g. CD28, OX40 and 4‐1BB) which improves cell proliferation, survival, and the ability to secrete cytokines. The fourth and fifth generation of CARs further enhanced the efficacy of CAR‐T cells by modifying transcription factor genes or additional costimulatory ligands. CAR's structure is still evolving and recent evidence shows slight changes in CAR structure can affect the activity of CAR‐T cells. For example, utilizing fully humanized scFv can improve the function of CAR‐T cells by reducing immunogenicity and increasing anti‐tumor activity.^[^ ^36^ ^]^ Short scFv linkers can drive receptor homodimerization and promote intracellular signal transduction, contributing to enhanced anti‐leukemia effect compared to long scFv counterparts.^[^ ^37^ ^]^ Additionally, modulating the intracellular signaling can affect CAR‐T activity. For example, mutating specific sites in the CD28 domain can benefit the safety and persistence of CAR‐T cells.^[^ ^38^ ^]^ CD3ζ truncated signaling containing only one immunoreceptor tyrosine activation motif showed better anti‐tumor effect and persistence in animal models.^[^ ^39^ ^]^ Further, modifying the hinge and transmembrane regions can affect the signal transmission and regulate cytokine secretion of CAR‐T cells.^[^ ^40^ ^]^

Currently, CAR‐T therapy has achieved remarkable clinical achievement. The first FDA approved CAR‐T therapy is used for B‐cell malignancies treatment. Such hematological tumors are contributed by the malignant transformation of pre‐B cells in the bone marrow and are featured by the high expression of the B cell lineage surface marker CD19. Next to that, the FDA approved three other CAR‐T cell drugs targeting CD19 to treat of ALL or diffuse large B‐cell lymphoma (DLBCL). Similar to TCR‐T cell therapy, patients with ALL may develop resistance due to the loss of the antigenic epitope on CD19.^[^ ^41^ ^]^ Therefore, tandem CARs targeting CD19/CD22 or CD19/BCMA have been developed to prevent cancer escape.^[^ ^42^ ^]^ In addition, CAR‐T therapies based on other surface antigens of malignant tumors, such as synovial sarcoma X breakpoint 2 (SSX‐2), Mesothelin (MSLN), B‐cell maturation antigen (BCMA) and CD123, are also underway in clinic.^[^ ^43^ ^]^ For example, clinical trials are currently exploring the potential of CAR‐T therapy that targets mesothelin as a treatment option for malignant pleural mesothelioma.^[^ ^44^ ^]^ Early results from the trial have shown some promising results, with 8/18 patients achieving stable disease or partial responses.

Though promising in hematological disease treatment, the efficiency of CAR‐T in solid tumors is below expectation.^[^ ^45^ ^]^ Due to the heterogeneity of tumor markers, attempts have been made to explore the ideal target antigens on solid tumors such as anti‐human epidermal growth factor receptor 2 (HER2), which is overexpressed in breast cancer. However, clinical trials showed that CAR‐T cells targeting HER2 could cause severe pulmonary toxicity due to the cross‐reaction on the lung epithelium (NCT02442297). More recently, CAR‐T cells targeting glypican‐3 (GPC3) were reported to treat GPC3‐positive hepatocellular carcinoma (HCC) in clinical trials (NCT02715362, NCT03198546, and NCT02905188). Other CAR candidates such as prostate‐specific membrane antigen (PSMA) targeting prostate cancer; mucin1 (MUC1) targeting non‐small cell lung cancer; MSLN, anthrax toxin receptor 1 (anthrax toxin receptor 1, ANTXR1) and MUC3A targeting gastric adenocarcinoma; epidermal growth factor receptor variant III (EGFRv III) and IL13R2 targeting glioblastoma (GBM) have shown promise in preclinical studies. In addition to exploring target antigens, developing multi‐targeting antigen CARs is another way to overcome antigen heterogeneity in tumor tissues. For example, CARs targeting HER2/IL13Ra2 and HER2/MUC1 were used for glioblastoma and breast cancer treatment, respectively.^[^ ^46^ ^]^ These dual‐targeting CAR‐T therapy exerted superior anti‐tumor effect compared to single‐targeting CAR‐T cells. Apart from identifying appropriate targets, researchers are also exploring ways to increase the specificity and activity of CAR T cells by developing CAR‐γδ T cells. CAR‐γδ T cells are able to recognize a wide range of tumor‐specific‐antigens, penetrate solid tumors and resist the immunosuppressive microenvironment within tumors. These characteristics make them a promising candidate for treating solid tumors. For instance, an ongoing phase I clinical trial (NCT04003649) is currently examining the safety and effectiveness of CAR‐γδ T cells that target the folate receptor alpha (FRα) antigen, with a focus on patients with solid tumors that have not responded to traditional treatments, such as ovarian and pancreatic cancers.

Besides tumor heterogeneity, another challenge of CAR‐T therapy in solid tumor treatment is the tumor immunosuppressive microenvironment. Programmed cell death 1 (PD‐1)/programmed cell death ligand 1 (PD‐L1) signaling represents an important immunosuppressive pathway that can induce T cell dysfunction and exhaustion. Therefore, researchers are exploring various strategies, such as combining CAR‐T cells with PD‐1/PD‐L1 antibodies to inhibit the PD1/PD‐L1 signaling‐induced immunosuppression.^[^ ^47^ ^]^ In addition to inhibiting PD‐1/PD‐L1 signaling, the local administration or genetic modification of CAR‐T cells with chemokines, such as CXCR2 or CXCR1, can enhance the migration and infiltration of CAR‐T cells into solid tumors.^[^ ^48^ ^]^ Enzymes‐engineered CAR‐T cells are also being developed to degrade the extracellular matrix of tumor stroma for improved infiltration and anti‐tumor effect. For instance, heparinase‐engineered and fibroblast activation protein (FAP)‐targeted CAR‐T cells have been engineered to degrade the heparin sulfate proteoglycan (HSPG) and fibroblast respectively which displayed improved infiltration and antitumor effect in the pre‐clinical studies.^[^ ^49^ ^]^ Additionally, CAR‐regulatory T cell (Treg cell) therapy is another approach to overcoming the tumor's immunosuppressive microenvironment, by targeting and suppressing myeloid–derived suppressor cells (MDSCs)and Tregs and enhancing the activity of conventional T cells to promote an anti‐tumor immune response.^[^ ^50^ ^]^ For example, CAR‐Treg cells can target and suppress the activity of MDSCs, which can impair the function of T cells and promote tumor growth.^[^ ^51^ ^]^ By specifically targeting and suppressing Tregs, CAR‐Treg cells can be optimized for better anti‐tumor activity than conventional T cells.^[^ ^52^ ^]^

Currently, Researchers are committed to advancing CAR‐T cell therapy in solid tumor treatment, with a focus on improving its trafficking and infiltration. More efficient and standardized methods for CAR‐T cell manufacturing are being developed to enhance its scalability and accessibility. Moreover, the identification of novel cancer antigens and the use of personalized medicine approaches are expected to increase the specificity and effectiveness of CAR‐T cell therapy.

### 2.4 Chimeric antigen receptor‐engineered natural killing cells (CAR‐NK cells)

Following the success of CAR‐T cells in clinical trials, ACT using other immune cells, such as natural killer (NK) cells, has attracted widespread interest. With their ability to rapidly identify and eliminate tumor cells, as well as their potential for CAR modification, NK cells are an appealing option for ACT.^[^ ^6a^ ^]^ One advantage of CAR‐NK cells is their relatively low toxicity compared to CAR‐T cells. NK cell activation is independent of the MHC‐mediated pathway, which eliminates concerns about GVHD.^[^ ^53^ ^]^ Besides, the cytokines produced by NK cells, including IFN‐γ and granulocyte‐macrophage colony‐stimulating factor (GM‐CSF), are less likely to cause cytokine release syndrome (CRS) and neurotoxicity. Moreover, CAR‐NK cells can inhibit cancer cell growth through natural pathways, including receptor‐stimulatory pathways, such as CD226 and killer immunoglobulin‐like receptors (KIRs), and antibody‐dependent cytotoxicity (ADCC). This gives CAR‐NK cells the potential to treat tumor cells that lack the targeted antigen of the CAR.

The source of NK is not limited to autologous NK cells in peripheral blood and umbilical cord blood. CD56^+^CD3^−^ tumor‐derived NK92 cell lines and pluripotent stem cells (iPSCs) have been reported to construct CAR‐NK cells.^[^ ^54^ ^]^ Among them, NK92 cells are most used because they can proliferate indefinitely *ex vivo*. More recently, iPSCs‐derived NK cells are extensively studied due to their ease of genetic engineering. As for CAR construction, CARs designed for T cells can be applied for NK cells. Commonly used CARs are composed of exocellular domains targeting CD19, HER‐2, EGFR, EGFRvIII, CS1, NKG2D etc. with costimulatory domains such as CD28, 4‐1BB and NK‐specific co‐stimulatory domain 2B4.^[^ ^55^ ^]^ For example, a Phase I/II clinical trial of CD19‐targeting CAR‐NK therapy showed that 63.6% (7/11) of patients with relapsed/refractory CD19^+^ NHL or chronic lymphocytic leukemia achieved complete response and there was no neurotoxicity or cytokine storm observed.^[^ ^56^ ^]^ In addition to CD19, clinical studies of CAR‐NK therapy targeting CD22 (NCT03692767), BCMA (NCT05008536, NCT03940833) CD33 (NCT02944162), or CD7 (NCT02742727) are also ongoing for hematopoietic malignancies treatment. CAR‐NK therapy has also been used to treat glioblastoma, ovarian cancer, hepatocellular carcinoma, and prostate cancer. HER2‐targeting CAR‐NK cells have been found to be effective in treating mouse glioblastoma^[^ ^57^ ^]^ and a phase I clinical trial is ongoing (NCT03383978). Another phase I clinical trial (NCT03692637) showed that CAR‐NK cells targeting ovarian cancer expressing MSLN displayed specific anti‐tumor activity both in vitro and in vivo. To circumvent tumor immunosuppressive microenvironment, CAR‐NK cells containing CD28/CD137 signaling domains coupled with a truncated PD‐1 peptide were developed to target and kill MUC1‐positive cells which are abundant in metastatic solid tumors. A phase I clinical trial showed that these MUC‐1‐NK cells can be effective in different solid tumors and none of the patients experienced cytokine storm or myelosuppression (NCT02839954).

Overall, clinical trials have displayed encouraging results in treating multiple tumors using CAR‐NK cells targeting various antigens. However, there is still a need for further research to optimize CAR‐NK cell production, persistence, and efficacy, as well as to identify the most effective antigen targets and combination therapies. It is also important to carefully evaluate the safety profile and potential long‐term effects of CAR‐NK cell therapy. With the growth of CAR‐NK cell technologies, this therapy is expected to become an important addition to other ACT for cancer treatment.

### 2.5 Chimeric antigen receptor‐engineered macrophages (CAR‐macrophages)

Inside the tumor microenvironment, tumor‐associated macrophages (TAMs) are the most abundant immune cells where they can promote tumor development and mediate immunosuppression.^[^ ^58^ ^]^ While inhibiting or depleting TAMs is generally used to reprogram the tumor microenvironment, the phagocytic and penetration ability of TAMs spark interest in constructing TAMs as therapeutics. In this context, CAR‐macrophages were developed for cancer treatment. One outstanding characteristic of CAR‐macrophages is their ability to infiltrate into tumor tissues, making CAR‐macrophages advantageous in treating solid tumors. There are a couple of pre‐clinical trials examining CAR‐macrophages’ ability to treat solid tumors.^[^ ^59^ ^]^ For example, phagocytes CAR‐macrophages and HER2 targeting CAR‐macrophages were used to treat liver cancer and breast cancer respectively.^[^ ^60^ ^]^ Another feature of CAR‐macrophages is that they can respond to the tumor environmental stimuli and transit into a favorable phenotype against immunosuppressive TME. For example, CAR‐expressing iPSC‐induced macrophage (CAR‐iMac) cells were designed with the capacity to tune the phenotypes in response to antigens.^[^ ^61^ ^]^ In the absence of antigens, CAR‐iMacs are closer to the M2 phenotype. However, when encountering the specific antigens on leukemia and lymphoma cells, CAR‐iMacs can convert to the pro‐inflammatory M1 state through CAR‐mediated signaling. CAR‐iMacs in this state can rapidly expand and exert a long‐lasting anti‐tumor effect in vivo.

One concern with CAR‐macrophage‐based therapy is that macrophages can only engulf fragments of targeted cells. To address this limitation, the combination of antiCD47 antibody and CAR‐macrophages were used to facilitate the phagocytosis of whole cells.^[^ ^62^ ^]^ Another strategy is introducing engulfment receptor intracellular domains into CAR construct to trigger engulfment of whole cells. For example, CAR containing Megf10, ɣ subunit of Fc receptors (chimeric antigen receptor‐phagocytes, CAR‐Ps) was engineered to macrophages to trigger phagocytosis of the cell expressing specific antigens.^[^ ^60a^ ^]^ Further, CAR‐Ps were linked with PI3K p85 subunit to construct a “tandem” CAR (CAR‐Ptandem) that can engulf human cancer cells effectively.^[^ ^60a^ ^]^

Despite the promising anti‐tumor application prospects reported in preclinical studies, there is only one ongoing clinical trial of CAR‐macrophage for the treatment of HER2‐positive breast cancer (NCT04437733). This clinical trial aims to evaluate the safety and feasibility of administering CAR‐macrophages to patients with HER2‐positive solid tumors with limited treatment options and poor prognoses, such as breast, gastric, and lung cancers. In addition, the manufacturing process for CAR‐macrophage is also a challenge. CAR‐macrophage is typically prepared through viral transfection, which may cause insertional errors, and the manufacturing process can be time‐consuming. To overcome these challenges, researchers are exploring alternative methods for generating CAR‐macrophages, such as genome editing and non‐viral delivery systems. Further research is needed to optimize the manufacturing process to improve its clinical feasibility.

### 2.6 Dendritic cell (DC) vaccines

In cancer immunotherapy, adoptive DC transfers have been investigated as cancer vaccines.^[^ ^63^ ^]^ This involves isolating and expanding autologous DCs in vitro, loading them with antigens, and returning them to patients. DC vaccines have demonstrated their ability to prompt a targeted and potent anti‐cancer immune response in various immune cells. Sipuleucel‐T, a DC vaccine that uses recombinant fusion protein antigens such as prostatic acid phosphatase (PAP) and GM‐CSF, is currently the only clinically approved DC vaccine for the treatment of prostate cancer.^[^ ^64^ ^]^ Studies have shown that it can stimulate a systemic immune response when infused in patients with metastatic castration‐resistant prostate cancer (mCRPC).^[^ ^65^ ^]^ Other clinical trials have investigated the combination use of DC vaccines with chemotherapy or immune checkpoint inhibitors.^[^ ^66^ ^]^

The collection and *ex vivo* manipulation of DCs is critical to the immune effect of DC vaccines. Generally, the source of DCs can be monocyte/progenitor cells derived cells or naturally occurring DC subsets. The former has been widely used clinically.^[^ ^67^ ^]^ Autologous DCs derived from CD14^+^ monocytes or CD34^+^ progenitor cells demonstrated clinical safety and potential efficacy against melanoma, prostate cancer and metastatic CRC.^[^ ^68^ ^]^ Moreover, due to higher MHC expression, naturally occurring DC subsets have greater antigen‐presenting capacity and clinical promise. For example, one clinical trial used blood DCs from melanoma patients which were expanded, harvested, activated, and loaded with CT antigens. Such DC vaccines induced significant antigen‐specific immune responses.^[^ ^69^ ^]^ Other DC subsets such as pDCs and/or cDC2s are under clinical trials for safety and efficiency validation (NCT02993315, NCT02692976, NCT02574377, NCT03747744 and NCT03707808). However, collected DCs may be dysfunctional *ex vivo* and the availability of DCs can be an issue due to their small cell proportion. Therefore, DC activation and stimulation are generally used to address these concerns.

As for the *ex vivo* manipulation, antigen loading, and DC maturation/activation are important for the appropriate function of DC vaccine. The selection of antigens is generally dependent on the therapeutic purpose and targeted tumors. For example, several clinical trials transferred neoantigen‐loaded DCs to patients with melanoma which generated a variety of neoantigen‐specific T cells NCT03300843, NCT03674073 and NCT01885702). To promote the internalization of antigen to DCs, DC targeting antibodies were used to modify the antigens. These antibodies coupled with antigens can facilitate cross presentation and immune responses. The significance of DC maturation/activation has been emphasized by several early clinical studies.^[^ ^70^ ^]^ Activation of DCs by cytokines, pathogen‐associated molecular patterns (PAMPs) and damage‐associated molecular patterns (DAMPs) or their combination has been used to facilitate DC migration and the subsequent immune response. However, the properties of these adjuvants and activators should be tuned for each DC subsets in the therapeutic context.

DC vaccines have shown great potential in the treatment of cancer by utilizing the critical roles that DCs play in antigen processing and presentation. Current research focuses on the development of cancer vaccines that involve the *ex vivo* manipulation of autologous DCs to induce anti‐cancer responses. However, the manufacturing process and administration of DC vaccines are still being refined, and ongoing research aims to enhance our understanding of the mechanisms of action and limitations of this approach. Moreover, optimizing the source of DCs, antigen selection, and DC maturation/activation are critical for the therapeutic effect of DC vaccines.

## 3 CHALLENGES AND OPPORTUNITIES OF ACT

### 3.1 Adoptive cell trafficking and infiltration

Generally, cell trafficking and infiltration in solid tumors remain a challenge, especially for adoptive T cells. Due to the abnormal vascular system, only a small portion of immune cells can migrate and infiltrate into the tumor tissues after systemic administration. In addition, down‐regulated expression of chemokines and adhesion molecules can inhibit the motility of immune cells.^[^ ^71^ ^]^ Therefore, allowing more immune cells to enter and penetrate the dense fibrotic stroma of solid tumors is the key to unleashing full potential of ACT therapy. One straightforward approach is to deliver adoptive cells through local administration. Intracranial, intrahepatic and intrapleural administration have been used to improve the therapeutic effect of ACT on glioblastoma liver metastases and pleural malignancies respectively.^[^ ^72^ ^]^ These studies generally showed that locoregionally administration improved the therapeutic effect compared with systemic injection. An alternative approach is to develop an effective cell delivery system. Biopolymers and nitinol films have been used as a scaffold to load CAR‐T cells to improve cell infiltration at tumor sites.^[^ ^73^ ^]^ These engineered delivery systems can also serve as a platform to co‐deliver the adoptive cells and other immunotherapeutic agents, thereby enhancing anti‐tumor immune response.

In addition to optimizing cell delivery approaches, other studies have modulated chemokines, cytokines, and growth factors in the tumor microenvironment to improve tumor infiltration. For example, some specific chemokines such as CCL2, CCL3, CCL4, CCL5, CXCL9 and CXCL10 are upregulated at the tumor sites with high T cell infiltration.^[^ ^48d^ ^]^ Modifying immune cells with the aforementioned chemokine‐specific receptors can facilitate infiltration in tumor tissues. In addition, cytokines such as IL‐10, TGF‐β, G protein signaling 5 (RGS5) or anti‐vascular endothelial growth factor (VEGF) hinder immune cells from infiltrating into tumor tissues.^[^ ^74^ ^]^ Combination use of small molecule inhibitors or engineering adoptive cells with functional peptides to inhibit the cytokine‐based pathway are another approach to improve cell infiltration. For example, in an animal model of pancreatic islet duct adenocarcinoma, the use of IL‐15‐activated NK cells, CD40 monoclonal antibody, and GM‐CSF‐secreting vaccines can promote immune cell infiltration by partially digesting the fibrous stroma in the tumor microenvironment. Similarly, inhibition of protein tyrosine kinase 2, which is involved in stromal fibrogenesis, prolonged the survival of tumor‐bearing mice, and made them more sensitive to ACT and PD‐1 therapy. However, it is noted that cytokine interactions are complex, and their safety and efficacy should be carefully examined.

### 3.2 Tumor immunosuppressive microenvironment (TME)

TME is a complex and dynamic system that has a crucial impact on cancer progression and immune response to ACT. To overcome TME, targeting the physical properties of the TME has been proposed, including abnormal vasculature and hypoxia. One approach to improve the physical properties of the TME is using vascular normalization agents, which can reduce abnormal vasculature and improve blood flow in tumors. This can increase the delivery of adoptively transferred T cells and other therapeutic agents, as well as improve the penetration of oxygen and other nutrients into the tumor microenvironment. The researchers have used anti‐angiogenic agents to normalize the tumor vasculature and reduce hypoxia, which can limit T cell activity.^[^ ^71a^ ^]^ In addition, strategies that target hypoxia, such as hypoxia‐activated prodrugs or HIF inhibitors, may also be useful in improving the efficacy of ACT.^[^ ^45^ ^]^ By reducing the accumulation of immunosuppressive cells and molecules, these approaches can improve the activity of adoptively transferred T cells.

Also, it is important to promote immune cell infiltration that is hindered by the immunosuppressive TME. Immune suppressor cells, including Treg cells,MDSCs, TAMs, are among the significant mechanisms responsible for immune evasion. These cells promote tumor growth by inhibiting the activation and function of effector T cells and suppressing the immune response.^[^ ^75^ ^]^ They can produce cytokines to shape the TME and weaken adoptive cell function. There are generally two strategies to overcome the tumor immunosuppressive environment. One approach is remodeling the tumor microenvironment by targeting suppressive cells. For example, MDSCs that overexpress natural killer group 2D (NKG2D) ligands can be depleted by CAR‐NK cells engineered with NKG2D receptors.^[^ ^76^ ^]^ Similarly, TAMs depletion can be achieved by stimulating factor 1 receptor (CSF1R) inhibitors.^[^ ^77^ ^]^ Interestingly, it was reported that reprograming TAMs agonistic to a M1‐like phenotype could yield a better anti‐tumor effect when combined with CAR‐T therapy.^[^ ^78^ ^]^ Another approach is to target immunosuppressive molecules. The immune checkpoint PD‐1/PD‐L1 signaling axis could be the most appreciated targeting pathway. The highly expressed PD‐L1 in tumors can bind to PD‐1 on the surface of immune cells, limiting immune cell activation and inducing its exhaustion. Currently, checkpoint inhibitors PD‐1 or PD‐L1 monoclonal antibodies are widely used in combination with ACT treatment. Other typical immune suppressive cytokines such as TGFβ, VEGF, IL‐4 and IL‐10 cannot only impact the immune cell function but also recruit the immune suppressive cells. In order to inhibit these cytokines based signaling, adoptive cells can either be engineered with receptors (e.g. TGF‐β receptors, IL‐4 receptors) or designed to release pro‐inflammatory cytokines (e.g. IL12, IL18 and IL 23) to boost the anti‐tumor immunity.^[^ ^78^, ^79^ ^]^

### 3.3 Safety considerations

The clinical success of ACT in the neoplastic hematologic disorder largely depends on the universal expression of the antigen CD19 and clinically manageable toxicity. ACT can lead to “on targeted/off‐tumor” toxicity contributed by the cross recognition of antigens expressed on both tumor and healthy tissues.^[^ ^80^ ^]^ Certain clinical trials have highlighted the cross‐reactivity of targeting antigens could cause lethal side effect.^[^ ^22^, ^24^, ^81^ ^]^ In order to explore more reliable targets, whole‐genome sequencing technology has been used to identify neoantigens of interest.^[^ ^82^ ^]^ However, considering the heterogeneity of antigen expression in the solid tumors, targeting one antigen may not be able to ensure the desired anti‐cancer outcomes. Therefore, multi‐targeting techniques such as bispecific CARs,^[^ ^83^ ^]^ trivalent CARs^[^ ^84^ ^]^ and bispecific T‐cell engager (BiTE)^[^ ^85^ ^]^ were developed to increase the tumor‐targeting specificity. In addition to multi‐targeting, Boolean AND‐gate logic was applied to engineer receptors which can trigger adoptive cell activation in a specific manner.^[^ ^86^ ^]^ A typical example is synthetic Notch (synNotch) receptor system which only induces CAR transcription after antigen recognition.^[^ ^87^ ^]^ This strategy is particularly applicable to tumors with high antigenic heterogeneity. A recent clinical has demonstrated the efficacy and safety of SynNotch‐CAR T in treating patients with glioblastoma.^[^ ^88^ ^]^ One limitation of the strategies described above is that the sophisticated design may increase the difficulty of large‐scale manufacturing. Recently, a completed phase I clinical trial used CRISPR‐Cas9 system to knock out the endogenous TCRs and PD1 genes of autologous T cells.^[^ ^89^ ^]^ These CRISPR edited T cells induced durable anti‐tumor effect with good tolerance in patients with refractory cancers. Therefore, precision gene‐editing technologies such as CRISPR‐based system can be a promising approach to navigate the production issues and may lead to ‘off‐the‐shelf’’ ACT products.

In addition to on‐target/off‐tumor toxicity, clinical data show that some patients treated with ACT can experience severe adverse events such as CRS. The management of CRS in patients receiving ACT involves several strategies, including supportive care and the use of drugs that target the underlying immune response. Supportive care measures aim to prevent further organ damage and manage any organ dysfunction caused by CRS. This includes administration of intravenous fluids, oxygen therapy, and vasopressors to maintain blood pressure. In addition to supportive care, drugs that target the underlying immune response have shown promise in managing CRS. Tocilizumab, a monoclonal antibody that blocks the IL‐6 receptor, has been approved by the FDA for the treatment of CRS in patients receiving ACT. Other drugs that have been used to manage CRS in ACT patients include corticosteroids, which have anti‐inflammatory properties and can reduce the production of inflammatory cytokines, and anakinra, an IL‐1 receptor antagonist that can also modulate the immune response.

## 4 CONCLUSIONS AND FUTURE DIRECTIONS

ACT has emerged as a groundbreaking approach to cancer immunotherapy and has rapidly advanced in recent years. It involves the transfer of immune cells with specific antitumor properties, such as TILs and CAR‐T cells, into patients to target and kill cancer cells. Other cell types, including NK cells, macrophages, DCs, and iPSC‐derived cells, are also being investigated as potential candidates for ACT. As summarized in Table 1, each type of ACT has its own set of advantages and limitations. Despite the challenges, researchers continue to explore and refine these therapies to improve their efficacy and safety, and to extend their applicability to a broad range of cancer types.

**TABLE 1:** Comparison of different ACT.

ACT Type	Advantages	Disadvantages
TIL	High response rate in melanoma; proven clinical efficacy	Limited availability and viability of TILs; complex and time‐consuming manufacturing process
TCR‐T	Broad applicability across different cancer types; potential for improved specificity and reduced toxicity compared to CAR‐T	Limited antigen specificity; risk for TCR mispairing; complex manufacturing process
CAR‐T	High response rate in hematologic malignancies; potential for sustained antitumor activity	Risk of cytokine release syndrome; limited persistence and expansion in vivo; complex manufacturing process
CAR‐NK	Lower cases of cytokine release syndrome compared to CAR‐T; potential for improved safety profile	Limited persistence and expansion in vivo; potential for low specificity
CAR‐macrophage	Potentially penetrate solid tumors; high phagocytic activity	Limited clinical data; immunosuppressive effects
DC vaccine	Stimulate a broad immune response; potential for enhanced T cell memory	Complex manufacturing process; limited antigen specificity

One major direction of ACT research is the development of new cell subsets to increase the enrichment of cells in solid tumor tissues.^[^ ^90^ ^]^ Furthermore, personalized medicine approaches, such as identifying patient‐specific neoantigens, may further enhance the specificity and efficacy of ACT. Another direction of ACT research is the development of combination therapies that combine ACT with other cancer treatments, such as immune checkpoint inhibitors, chemotherapy, and radiotherapy. These combination therapies have the potential to enhance the efficacy of ACT and overcome the challenges posed by the TME.

Efforts are also underway to improve the scalability and accessibility of ACT. This includes the development of more efficient and standardized methods for cell manufacturing, as well as the exploration of new delivery methods, such as nanocarriers, to improve the delivery of adoptively transferred cells to the tumor site. However, one significant production challenge with ACT is its cost, which can be substantial due to the complexity of the manufacturing process and the personalized nature of the therapy. This cost can limit access to ACT for many patients who may not have access to the necessary resources or insurance coverage. Therefore, efforts to improve the scalability and cost‐effectiveness of ACT are essential to make it more accessible to a broader range of cancer patients. In vivo editing of lymphocytes is a promising approach which may eliminate the need for the costly and time‐consuming process of manufacturing *ex vivo* and allows for the rapid adaptation of the therapy to changing tumor conditions. While pre‐clinical studies have shown the potential of CRISPR systems for in vivo editing of CAR‐T therapy,^[^ ^91^ ^]^ further research is required to optimize delivery systems, enhance the safety profile, and evaluate the long‐term effects of editing immune cells in vivo.

Overall, the ongoing development of ACT highlights its potential as a therapeutic approach for treating various diseases beyond cancers.^[^ ^92^ ^]^ More clinical trials are expected to provide in‐depth information and advance various types of ACT as a safe and effective therapy.

## CONFLICT OF INTEREST STATEMENT

Yizhou Dong is a scientific advisory board member of Oncorus Inc, Arbor Biotechnologies, and FL85. Other authors declare no conflicts of interest.
